# Implementation research in Primary Care (Part 1): What is implementation research?

**DOI:** 10.51866/cm.1067

**Published:** 2026-01-31

**Authors:** Chor Yau Ooi, Wen Ting Tong, Anne Sales, Chirk Jenn Ng

**Affiliations:** 1 Department of Family Medicine, Faculty of Medicine and Health Sciences, Universiti Malaysia Sarawak, Jalan Datuk Mohammad Musa, Kota Samarahan, Sarawak, Malaysia.; 2 Department of Primary Care Medicine, Faculty of Medicine, Universiti Malaya, Kuala Lumpur, Malaysia.; 3 Sinclair School of Nursing and Department of Family and Community Medicine, MU School of Medicine, University of Missouri, Missouri, Columbia, MO 65211, USA.; 4 Department of Research, SingHealth Polyclinics, Singapore.; 5 Centre for Population Health Research and Implementation, SingHealth Regional Health System, Hospital Boulevard, #19-01 SingHealth Tower, Singapore.

**Keywords:** Implementation science, Primary health care, Translational research, Internet-based intervention, Shared decision making

## Abstract

Implementation research plays a vital role in narrowing the “evidence-to-practice” gap in primary care by ensuring that evidence-based interventions are not only adopted but also embedded into routine clinical practice in a sustainable way. This is especially important in primary care, where clinicians care for diverse patient groups, work within evolving healthcare systems, and face rapid technological change. Implementation research is defined as *“the scientific study of methods to promote the systematic uptake of research findings and other evidence-based practices into routine practice, thereby improving the quality and effectiveness of health services.”* This commentary introduces the concept and significance of implementation research, outlines its place within the broader research translation continuum, and clarifies how it differs from quality improvement. It also provides examples of how implementation research is applied in primary care to support the adoption of new innovations.should be further verified.

## Why implementation research?

Despite groundbreaking advances in biomedical research, it takes 17 years for only 14% of research findings to impact patient care.^1^ This delay costs billions,^2^ and undermines healthcare outcomes; only 60% of persons in healthcare settings receive care according to evidence-based guidelines.^3^ There are various reasons why it is difficult to move an evidence-based intervention from a clinical research environment to the real-world health services. In the research world, evidence-based innovations are often implemented under controlled conditions to maximise its efficacy whereby participants were carefully selected, and specialized and trained researchers were the ones who conduct the implementation and evaluation, and all these are supported by research funds. But in the real-world health services practice, the intention for implementation is to achieve sustainable delivery and widespread adoption without selecting the target population. Also, implementers are usually clinicians with no specialized training on the implementation, and there is often limited funds to deliver these services. The issues highlighted above have prompted healthcare funders, policymakers and researchers to act. Hence, the emergence of the field implementation research, which aims to close the research-practice gap. Implementation research directly tackles implementation barriers by identifying obstacles and designing strategies to ensure the sustainable adoption of proven interventions.

## The Role of Implementation Research in Primary Care

Implementation research is essential for bridging the “evidence-to-practice” gap in primary care, ensuring evidence-based interventions are not only adopted but also integrated into everyday clinical settings in a sustainable manner. This is crucial in primary care, where practitioners have to manage diverse patient populations, adapt to evolving healthcare systems, and navigate rapidly changing technologies.^4^

Primary care often requires the implementation of complex interventions tailored to dynamic and varied contexts. Despite strong evidence supporting patient-centred approaches and interventions that improve outcomes, significant barriers—such as resource constraints, policy limitations, and organizational differences—frequently hinder their integration into routine practice.^5^ Implementation research identifies these challenges and provides structured strategies to overcome them systematically.

Using frameworks such as Reach, Effectiveness, Adoption, Implementation, and Maintenance (RE-AIM), and the Consolidated Framework for Implementation Research (CFIR), implementation research supports the development, adoption, and sustainability of interventions by aligning them with the unique needs, resources, and contexts of specific primary care settings.^4^ This ensures that interventions are practical, effective, and adaptable over time. By focusing on systematic change and multidisciplinary insights, implementation research helps optimize workflows, enhance patient satisfaction, and improve health outcomes.^4^

Ultimately, implementation research empowers primary care practitioners to transform evidence-based research into actionable, sustainable practices, enabling high-quality, patient-centred care that addresses real-world challenges in an evolving healthcare landscape.

## What is Implementation Research?

Implementation research is defined as *“the scientific study of methods to promote the systematic uptake of research findings and other evidence-based practices into routine practice, thereby improving the quality and effectiveness of health services”.^6^* In simpler terms, it means the promotion of adoption and integration of evidence-based practices, interventions, and policies in routine practice. The goal of implementation research is not to establish the health impact of a clinical innovation (i.e., efficacy research), but rather to identify the factors that affect its uptake into routine use.^7^ Implementation research also focuses on understanding the behaviour of healthcare professionals and other stakeholders as a key variable in the sustainable uptake, adoption, and implementation of evidence-based interventions.^6^

The scope ofimplementation research includes exploring barriers and facilitators to implementation; exploring influences on the provider, patient, and organizational behaviours; developing and testing the effectiveness of implementation strategies; determining implementation adaptation and fidelity, mechanisms of impact, and contextual influences on implementation and outcomes, sustainability, and scalability; developing or expanding theories and frameworks to support implementation; and developing and validating measures to assess implementation outcomes.^8^

Curran et al., (2020) develops a term to provide a clearer understanding on the definition of implementation research, and a description of “its place” among related fields.^9^ The term describes the evidence-based innovation of interest as “the thing”. Implementation research aims to study how to get people or organisation to do “the thing”, or what implementation strategies or the “stuff we do” to try to help people and organizations to do “the thing”. On the other hand, clinical efficacy and effectiveness research investigates whether “the thing” works.^9^

## Implementation Research within the Research Translational Continuum

Research translation is the process of translating research findings into practice and includes five distinct phases of research (T0-T5). T0 involves the conduct of basic science research to generate knowledge at molecular level. T1 is Phase 1 and Phase 2 clinical trials where T0 findings are moved to humans for testing of proof of concept to develop new methods of diagnosis, treatment or prevention. T2 is where research findings are translated to patients but tested in a more controlled environment, i.e., as Phase 3 clinical trial. In T3, findings from T1 and T2 are shared with the community, driving innovation in translating new clinical knowledge into practice through approaches such as health services, community-based participatory, and comparative effectiveness research. T4 is the translation of research findings into community where population level outcomes research is conducted to assess the true benefit of the innovation at the societal level.

**Figure 1:**
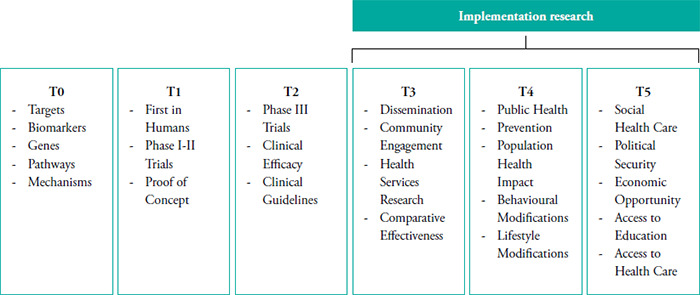
Implementation research within the research translational continuum (Adapted from: Clinical and Translational Science: From Bench-Bedside to Global Village)^11^

The Canadian Institutes of Health Research highlighted the main challenge in trying to translate research findings into practice was the lack capacity of researchers to synthesize, disseminate and integrate research results more broadly into health care decision-making and clinical practice.^10^ Implementation research addresses challenges in the T3 and T4 stages of the research translation continuum by bridging the gap between evidence generation and population health impact. In T3, it focuses on identifying barriers and facilitators to the adoption of evidence-based interventions, developing and testing strategies to improve their integration into real-world clinical and community settings, and ensuring their sustainability. In T4, implementation research supports the scale-up of effective interventions, evaluates their cost-effectiveness, equity, and long-term outcomes, and informs policy and system-level changes. Through these efforts, implementation research ensures that research findings are effectively translated into practice to improve health and population outcomes. In recent years, T5 has emerged, representing a shift toward a social health model, focusing on global wellness by addressing structural barriers such as poverty, inequity, instability, and malnutrition.^11^ It emphasizes equity and requires multidisciplinary collaboration beyond medicine. Implementation research aligns with T5 by providing methods to ensure proven interventions are adopted, scaled, and sustained. While T5 defines what must change at the societal level, implementation research explains how to make it happen through context-specific, sustainable strategies (Figure 1).

## The difference between Implementation Research and Quality Improvement

Implementation research and quality improvement have similar characteristics; both share the same goals of enhancing healthcare delivery and patient outcomes, rely on empirical evidence, and consider the importance of healthcare context in their approach. Implementation research and quality improvement also focus on iterative process in their approaches to understand healthcare processes and implement innovations into local context. Another overlapping characteristic of these approaches is the use of process models, framework such as Knowledge to Action Framework for implementation research, and Plan-Do-Study-Act for quality improvement. Both fields also adopt a common data collection approach including quantitative, qualitative, or mixed-methods approach. Due to these similarities, the distinction between implementation research and quality improvement is often blurred and confusing, causing researchers to be unsure of which approach to adopt in their implementation endeavours. To enable a better understanding of the characteristics of implementation research, Table 1 describes the differences between implementation research and quality improvement.

**Table 1:** Similarities and differences between implementation research and quality improvement

	Implementation research	Quality improvement
**Study aim**	To evaluate implementation strategies. Implementation strategy is the approach used to enhance the adoption, implementation, and sustainability of a clinical innovation. Example of implementation strategy are: training, audit-feedback, or incentives.	To improve clinical practice by targeting specific processes within a particular setting to enhance performance
**Intervention target**	Clinicians or organization practice	
**Primary outcome**	Rates on the use of the clinical innovation such as reach, adoption, and quality of use of the clinical innovation such as fidelity and sustainability. Findings are generalizable principles and methods for implementing evidence-based practices.	Findings are similar to implementation research primary outcomes but are context-specific and not generalisable
**Unit of analysis or randomisation**	Clinicians, team or facility	
**Conception of study**	Study begins with an innovation, and the aim is on how to implement the innovation in routine practice.^12^	Study begins with a problem in healthcare system, and the aim is on how to improve care processes to achieve a clinical priority goal.

## Examples of Implementation Research in Primary Care

Implementation research in primary care settings across diverse countries have demonstrated the utility of frameworks to evaluate, design, and optimise health interventions. These studies underscore the critical role of primary care in addressing global health challenges, highlighting both the promise of innovative programmes and the persistent gaps in implementation research.

In India, research on HPV-based cervical cancer screening has demonstrated the practicality and acceptability of self-collection methods. However, significant obstacles remain, particularly among underrepresented groups such as tribal and northeastern communities. These include stigma, logistical challenges, and inadequate follow-up mechanisms, which weaken the effectiveness of such programmes.^13^ Similarly, efforts to address diabetes and hypertension in rural areas have encountered difficulties, such as frequent staff transfers and resistance to task-sharing, despite initial improvements in workflow efficiency and patient follow-up. These insights underscore the need for sustainable models that account for contextual challenges, ensure programme continuity, and align with existing healthcare systems.^14^ In China, falls prevention programmes for older adults have been hindered by gaps in professional knowledge and limited financial incentives, even though they benefit from robust governmental support. These findings highlight the importance of tailoring interventions to specific contexts, fostering intersectoral collaboration, and relying on evidence-based planning to scale successful initiatives.^15^

In Southeast Asia, efforts to integrate public health interventions into primary care systems have revealed both opportunities and obstacles. In the Philippines, integrating COVID-19 vaccination into routine services demonstrated the potential for leveraging existing infrastructure to enhance equity and efficiency. However, vaccine hesitancy and logistical barriers limited the program’s reach.^16^ In Malaysia, studies on weight management, insulin patient decision aid, and mental health services highlighted systemic challenges, including resource constraints, clinician perceptions, and workload issues. Yet, they also revealed the power of tailored strategies, such as stakeholder engagement, interprofessional collaboration, and the use ofeHealth tools, to overcome these barriers.^17-20^ Meanwhile, in Indonesia, tuberculosis screening for diabetes patients in private clinics was found to be acceptable and feasible but hampered by limited resources, regulatory hurdles, and patient reluctance. These findings suggest the need for systemic approaches, such as integrating electronic medical records and fostering public-private collaboration, to enhance screening outcomes.^21^

Across these settings, a recurring gap in implementation research is the lack of follow-up data and sustainability measures. Many studies focus on initial implementation without adequately addressing long-term maintenance and scalability. Additionally, while frameworks like TICD and CFIR provide valuable insights into barriers and facilitators, their application often reveals gaps in capturing dynamic, real-world complexities, such as shifting stakeholder priorities and resource constraints. Future research should emphasize longitudinal designs, participatory approaches, and cross-sectoral integration to bridge these gaps and strengthen primary care systems globally.

Box 1 **Case Study: Developing and evaluating strategies to implement an insulin patient decision aid in an academic primary care clinic in Malaysia**

Patient decision aids (PDAs) are decision-making tools to facilitate shared decision-making; however, their routine use in clinical consultations is still lacking. At the Universiti Malaya Medical Centre, an insulin PDA has been developed to help patients with type 2 diabetes to make informed decision about insulin therapy. An implementation research was commenced to develop and implement an implementation intervention to effectively integrate the insulin PDA in routine clinical practice at the primary care clinic. The implementation research was guided by the process model Knowledge to Action framework, and consisted of three phases: Phase 1: Explore barriers to knowledge use; Phase 2: Select, Tailor, Implement Interventions; Phase 3: Evaluate outcomes.

In Phase 1: A qualitative exploration was conducted to identify PDA implementation barriers in Malaysian public healthcare settings. Unique and prominent barriers to the insulin PDA implementation were found in the Malaysian healthcare settings such as role boundary, the lack of continuity of care, the lack of SDM culture, language barrier, and patient literacy level. These findings were used to inform the development of the implementation intervention in Phase 2.^22-24^

In Phase 2, an implementation intervention was systematically developed using the tailored implementation approach, where strategies were identified and mapped to address specific barriers to the insulin PDA implementation. Initially, the multi-voting technique was conducted to prioritise Phase 1 barriers. Next, strategies were then identified from literature review, pragmatic suggestions from clinic stakeholders, and implementation taxonomies to overcome the prioritised barriers based on evidence. The implementation intervention was finalised through a clinic stakeholders meeting involving healthcare policy makers, doctors, nurses, and patients with type 2 diabetes. In Phase 2, 13 barriers were prioritised and they were related to healthcare providers’ (HCP) roles, patient’s characteristics and attitudes, and follow-up difficulties. Eleven strategies including training, feedback, PDA availability, and systematic documentation were integrated into an implementation intervention to address the barriers.^19^

In Phase 3, a mixed-methods evaluation was conducted to assess implementation outcomes guided by the ‘Reach’, ‘Adoption’, ‘Implementation’, and ‘Maintenance’ dimensions of the RE-AIM framework. The outcomes showed that for ‘Reach’, 88.9% of the doctors received PDA training and this was attributed to their self-motivation, mandated changes, and timing of the PDA workshop. The PDA reached 387 patients and was facilitated by the doctors who delivered the PDA to them and their own desire to know more about insulin. Barriers to reaching patients were their attitudes towards their health, and lack of interest to initiate insulin. Doctors’ adoption of the PDA was high (83.3%) and was attributed to their positive experience with the PDA use, its usefulness, and the training workshop effectiveness. Barriers to adoption were patients’ non-use of the PDA, availability of doctors in the clinic, and the lack of effectiveness of the strategy ‘Provide feedback’. Patients’ adoption was moderate with only 65.7% who read the given PDA. Among the reasons for not reading the PDA were a lackadaisical attitude towards their health, and perceived adequate knowledge about diabetes and insulin. The degree of ‘Implementation’ of the PDA varied for different tasks and was challenging for reasons such as the perception of unnecessary steps, the clinic’s appointment system, and nurses’ attitudes. Finally, for ‘Maintenance’, 80% of the doctors were willing to continue using the PDA due to its benefits.^25^

In conclusion, this study highlights a systematic process of developing PDA implementation intervention. When implementing PDAs, it is crucial to consider the healthcare culture and system. Focusing on implementation efforts such as training to improve providers’ knowledge and skills, organisational leaders’ support, and utilising a documentation system to facilitate follow-ups can lead to a higher reach and adoption of PDAs.

Box 2 **Case Study: Implementing a web-based app for men’s health screening in a Malaysian primary care setting**

This case study highlights the implementation of a novel web-based app (ScreenMen) for health screening in men. ScreenMen is a web-based health screening app designed to increase men’s participation in screenings in Malaysia. The implementation of ScreenMen was guided by the Knowledge-to-Action (KTA) framework, particularly its Action Cycle. The study systematically followed key phases of this cycle, beginning with Phase I, which involved exploring challenges in implementing web-based screening apps. In Phase II, exploration of barriers and facilitators affecting the implementation of ScreenMen was done. Based on these findings, Phase III focused on designing and tailoring implementation strategies to ensure they were practical and contextually suitable for primary care setting. In Phase IV, these strategies were piloted in a real-world primary care setting to evaluate knowledge use, feasibility, and acceptability.

In Phase 1, a scoping review aimed at identifying implementation strategies for web-based health screening apps was conducted. The findings revealed that few studies have systematically explored the implementation process, with most research emphasizing the effectiveness of these apps rather than their integration into healthcare systems. Among the key implementation strategies identified were training and education for healthcare providers, executive board involvement, the use of clinical champions, reminders, and eHealth integration.^26^

Phase 2 explored the barriers and facilitators to implementing the ScreenMen in three primary care settings through a qualitative study. Using the Tailored Implementation in Chronic Diseases (TICD) framework, the study identified 12 key barriers and 12 facilitators, covering areas such as awareness, skills, resource availability, and system integration. One unique barrier was the language barrier, which had not been addressed in previous implementation studies. Additionally, four clinic-specific determinants were identified: observability, domain knowledge, intention and motivation, and quality assurance systems. The findings highlight that while technical feasibility and accessibility can aid adoption, factors like healthcare provider workload, resistance to change, and lack of infrastructure can hinder successful implementation.

Phase 3 detailed the development of a tailored implementation intervention for ScreenMen, based on the barriers and facilitators identified in Phase II. The intervention included six key strategies: involving executive boards, mandate change, providing education and training, identifying and preparing champions, using ICT solutions, and audit and feedback mechanisms. The intervention was further refined in response to the COVID-19 pandemic, which increased the workload of healthcare providers and limited in-person interactions. As a result, digital solutions such as QR codes and remote training were emphasized. This tailored approach aimed to improve the feasibility and sustainability of ScreenMen within the primary care setting.

In Phase 4, a pilot evaluation of the ScreenMen implementation in a government primary care clinic was conducted using the Reach, Effectiveness, Adoption, Implementation, and Maintenance (RE-AIM) framework. The study found that QR codes and clinic champions were effective in increasing patient engagement, but healthcare providers faced challenges due to high workloads and limited time. While awareness of men’s health screening improved, overall adoption remained moderate, and mandate-driven approaches were ineffective. The COVID-19 pandemic further impacted implementation by reducing clinic visits and increasing provider burnout. Despite these challenges, the study demonstrated that tailored strategies and digital facilitation were key to enhancing the adoption of ScreenMen.^18^

This study demonstrated the importance of a systematic, contextually tailored approach guided by the KTA framework. The study highlighted key barriers and facilitators, emphasizing the need for provider training, executive support, and digital solutions to enhance adoption. While strategies such as QR codes and clinic champions effectively increased patient engagement, challenges such as high provider workloads and resistance to change persisted. The COVID-19 pandemic further underscored the necessity of flexible, technology-driven interventions. Despite moderate adoption rates, the study demonstrated that a tailored implementation approach can improve feasibility, acceptance, and sustainability, ultimately supporting increased men’s participation in health screenings.

Box 3 **Key points**

- Implementation research is the scientific study of methods to promote the systematic uptake of research findings and other evidence-based practices into routine practice, thereby improving the quality and effectiveness of health services.
- Implementation research is essential for bridging the “evidence-to-practice” gap in primary care, ensuring evidence-based interventions are not only adopted but also sustainably integrated into everyday clinical settings.
- Within the Research Translational Continuum, implementation research seeks to identify and address challenges in stages T3-T5; to understand and improve delivery of an innovation where its effectiveness has been established to improve clinical practice (T3), population health (T4) and beyond the public health model of care to the social health model (T5).
- Primary care implementation research shows promise across diverse contexts but faces unique barriers, that require more sustainable, context-sensitive, and system-integrated approaches.

## Conclusion

Implementation research is vital for closing the evidence-to-practice gap in primary care by promoting the adoption, integration, and sustainability of evidence-based interventions. Frameworks such as RE-AIM, CFIR, and KTA help to identify and address barriers, tailor strategies to local contexts, and enhance outcomes. Evidence from diverse settings shows promise but also highlights persistent challenges, particularly in sustainability and scalability. Moving forward, implementation research must emphasize context-sensitive, system-integrated, and participatory approaches to ensure lasting impact on patient care and health system performance.
